# Supplementary figures and images for: Determinants of Aedes mosquito larval ecology in a heterogeneous urban environment- a longitudinal study in Bengaluru, India
Source: PLoS Negl Trop Dis. 2023 Nov 8;17(11):e0011702. doi: 10.1371/journal.pntd.0011702 (PMC10659209; doi:10.1371/journal.pntd.0011702)

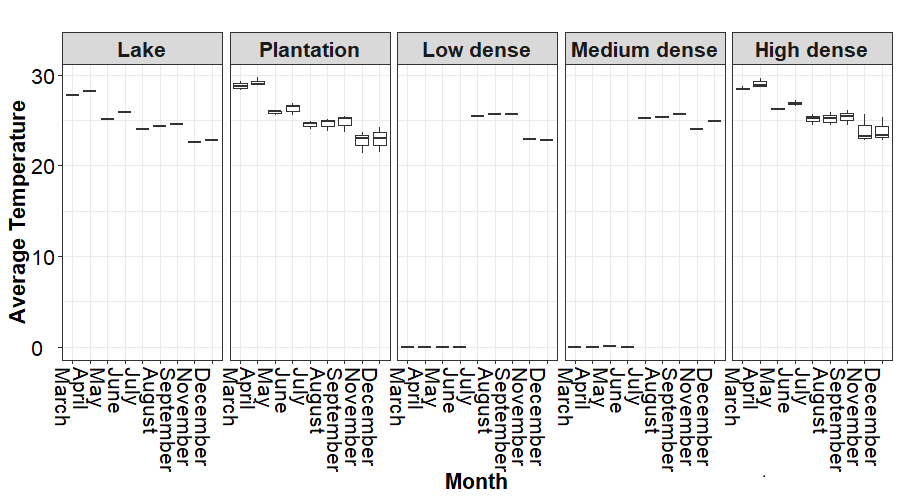

Supplement: S1 Fig — (TIF) [file pntd.0011702.s010.tif]
