# Supplementary material for: Determinants of Aedes mosquito larval ecology in a heterogeneous urban environment- a longitudinal study in Bengaluru, India
Source: PLoS Negl Trop Dis. 2023 Nov 8;17(11):e0011702. doi: 10.1371/journal.pntd.0011702 (PMC10659209; doi:10.1371/journal.pntd.0011702)
Supplement: Attachment — Submitted filename: Response_to_reviewers_ 26.09.2023.docx [file pntd.0011702.s012.docx]

To,

Drs. Roberto Barrera and Audrey Lenhart

Academic Editor and Section Editor

PLoS Neglected Tropical Diseases

Dear Editors,

Many thanks for constructive feedback on our manuscript. We have revised the manuscript as per suggestions by the two reviewers. Please find attached our point-by-point response to their comments.

I hope you will find this manuscript in order and consider it for publication.

Many thanks

Sincerely,

Farah Ishtiaq

(on behalf of co-authors)

*Key Review Criteria Required for Acceptance?
As you describe the new analyses required for acceptance, please consider the following:

Methods
-Are the objectives of the study clearly articulated with a clear testable hypothesis stated?
-Is the study design appropriate to address the stated objectives?
-Is the population clearly described and appropriate for the hypothesis being tested?
-Is the sample size sufficient to ensure adequate power to address the hypothesis being tested?
-Were correct statistical analysis used to support conclusions?
-Are there concerns about ethical or regulatory requirements being met?*

**Response to Reviewer 1**

*The methods used for this study are highly appropriate with an excellent use of mixed effect models; an appropriate way of determining key drivers of mosquito prevalence. The authors used models to inform and guide rather than provide a definitive set of predictors which is a welcome approach to such analysis.*

**Response: We thank the reviewer for positive feedback.**

*The methods are laid out well and clear for the most part, although the only minor comments are:
Line 200: a reference to the minimum distance between each grid could be made clearer – which grids are being referred to, either the Index grid or Random grid? I assume the Random grids, but it would be worth being explicit for the reader.*

**Response: We have clarified the sentence.**

**Line 202: The minimum distance between each grid (index and random) grids was approximately 300m-500m to maximum 1km.**

*Lines 201 – 204: It would be worth stating how long the ibuttons ran for. The whole of the sampling period or just one/two/more days? Likewise, it isn’t clear whether the ibuttons were deployed in the Index or Random grids, or indeed, both.*

# Response: Line 205: The loggers were deployed in completely shaded area and were set up to record at 30 min interval round -the- clock basis for the duration of the study.

**Response to Reviewer 2**

*Line 173:  The phrase that starts with "September being" is not a complete sentence.*

**Response:** **We have rephrased the sentence.**

**Line 171-173: Bengaluru experiences a semi-arid climate with temperature between 18°C to 34°C, and southwest monsoon season (June-September) with normal annual rainfall of 820mm which accounts for nearly 60% of the rainfall.**

*Line 184: Culicidae mosquito is redundant.*

**Response: Thank you for highlighting this. We have deleted this.**

*Lines 190, 191, 192 and thereafter:  density, not dense*

**Response: This has been changed throughout the manuscript.**

*Line 234: mosquitoes (plural)*

**Response: Line 237: We have corrected this.**

*Were data tested for normality prior to ANOVA?*

**Response: The ANOVA was conducted on mean species richness (species richness<- specnumber(mosq_abundance.matrix) and species diversity (Shannon<-diversity(mosq_abundance.matrix) obtained using vegan package -to test differences in species diversity measure across different habitat types. A square root transformation was applied to the data to decrease the influence of dominant species and the results were same as raw count, so we proceed with it.**

*Were the indices used in these calculations invented by the authors or were they derived from literature?*

**Response: Container index and pupal index were calculated according to WHO reference. The location and Breteau location index were calculated as per Troyo *et al.* 2008.**

*I like that the authors measured temperatures for both air and water inside container habitats.  This is an often-overlooked criterion.*

**Response: Thank you for your positive feedback.**

*For all statistical tests that are used, please include a short statement about reporting of results, for example, for ANOVA, "Results are reported as arithmetic mean +/- standard error."  This gives the reader an idea of what to expect.*

**Response: We have added this in line 283.**

--------------------

*Results
-Does the analysis presented match the analysis plan?
-Are the results clearly and completely presented?
-Are the figures (Tables, Images) of sufficient quality for clarity?*

**Response to Reviewer 1**

*The authors analysed the data completely as planned, and present a clear set of results.

There is a well presented data overview, covering core data before moving onto more complex analysis; each statistical analysis was correctly presented.*

*With the mixed model analysis, the authors kept to the key drivers/main models whilst presenting their results - I fully approve of this approach, as there is a tendancy to over-describe results from mixed models which can muddy the waters somewhat. Because of this approach, the results, particularly for the mixed effect models was clear and easy to follow.

Figures are of good quality and used to emphasise main findings. As such, the figures complement the writing and help enhance the narrative.*

**Response: We really appreciate your constructive feedback.**

**Response to Reviewer 2**

*Lines 371-373:  Were these species mentioned in full prior to this paragraph?  I must have missed that.* **Response: No, this is the first time here we mentioned these species recorded in the study.**

*I see chi-square values reported but I didn't find them mentioned in the methods section.  Same with beta values and Chao1 values.*

**Response: We report Wald’s χ2-tests-line 284 and Chao1 estimator -line 486.**

*Line 523:  This is not a complete sentence.  What am I supposed to understand from a half of a thought?*

**Response: Line 533: We have corrected this sentence.**

*Lines 387-388:  the authors mention no seasonal difference between BPR and they refer to Figure 3, but figure 3 doesn't give any information about seasonality of BPR.*

**Response: We have clarified this in Line 397 refers to Fig. 3 for BPR was significantly different in microhabitat.**

*Line 394:  reference for WHO thresholds?*

**Response: Line 403 refers to Breteau index based on WHO reference 16 and 22.**

--------------------

*Conclusions
-Are the conclusions supported by the data presented?
-Are the limitations of analysis clearly described?
-Do the authors discuss how these data can be helpful to advance our understanding of the topic under study?
-Is public health relevance addressed?*

**Response to Reviewer 1**

*The study has clear relevance for informing disease control from mosquito vectors. I completely concur with the conclusions drawn from the data.

The influence of the environment on mosquito breeding success, particularly those species linked with Dengue transmission, is clearly an important step in helping to control disease outbreaks. The study covered microhabitat and macrohabitat drivers and found that microhabitat, notably container presence (as well as season) helped determine mosquito presence and larval prevalence. This prevalence increased in urbanised areas, whilst species diversity decreased in urbanised areas leaving Aedes species as dominant species, clearly linked to dengue transmission.

The authors correctly state that "transmission reduction programmes should focus on ‘neighbourhood surveillance’" and within this context, reduction or removal of containers in urbanised areas would be a principal mechanism of reducing Aedes-borne diseases. The authors recognise that the link between dengue outbreaks and mosquito population dynamics is not clear, therefore further fine-scale data is required.

I can safely say that the relevance to public health is very clearly addressed.*

**Response: We are thrilled to see the reviewers’ positive comments on the study.**

**Response to Reviewer 2**

*I believe that the authors have drawn reasonable conclusions based on their work.*

**Response: Many thanks for your positive feedback.**
--------------------

*Editorial and Data Presentation Modifications?
Use  this section for editorial suggestions as well as relatively minor modifications of existing data that would enhance clarity. If the only modifications needed are minor and/or editorial, you may wish to recommend “Minor Revision” or “Accept”.*

**Response to Reviewer 1**

*As outlined above:
Line 200: a reference to the minimum distance between each grid could be made clearer – which grids are being referred to, either the Index grid or Random grid? I assume the Random grids, but it would be worth being explicit for the reader.*

**Response: We clarified the sentence.**

**Line 202: The minimum distance between each grid (index and random) grids was approximately 300m-500m to maximum 1km.**

*Lines 201 – 204: It would be worth stating how long the ibuttons ran for. The whole of the sampling period or just one/two/more days? Likewise, it isn’t clear whether the ibuttons were deployed in the Index or Random grids, or indeed, both.*

# Response: Line 205: The loggers were deployed in completely shaded area and were set up to record at 30 min interval round -the- clock basis for the duration of the study.

**Response to** **Reviewer 2**

*Some of the figures are a little hard to see - detail is lost at the small scale.  I wonder what the figures will look like in print.  I hope that they are readable.*

**Response: We will provide high quality figures. Thank you for your feedback**.
--------------------

*Summary and General Comments
Use this section to provide overall comments, discuss strengths/weaknesses of the study, novelty, significance, general execution and scholarship. You may also include additional comments for the author, including concerns about dual publication, research ethics, or publication ethics. If requesting major revision, please articulate the new experiments that are needed.*

**Response to Reviewer 1**

*I felt that this study was really well conducted, thought-out and analysed to a high standard. The relevance to public health is clear and with all studies obvioulsy drives a number of additional questions. The simplicity of the conclusions is fantastic - something that can be implemented at ground-level. It will be interesting to see how that message is communicated from the scientific literature to the relevant neighborhoods; I fear that communicating the message will be a harder task than the study.*

**Response: We appreciate reviewers’ concern. We have been working with local municipal bodies and citizen to communicate our findings.**

**Response to Reviewer 2**

*In the abstract, Line 78, Aedes albopictus =Stegomyia albopicta.  Aedes and albopictus are masculine but Stegomyia is feminine and so the ending must agree, ergo albopicta.*

**Response: Thank you for highlighting this. We have corrected this. Line 78.**
